# P47 Addressing the increasing burden of infection and AMR in older people: a propensity-score-matched case-control analysis of urinary tract infection management and outcomes in and outside care homes in England

**DOI:** 10.1093/jacamr/dlaf230.054

**Published:** 2025-12-04

**Authors:** Emma Carter, Raheelah Ahmad, Paul Aylin, Damien Ming, Tom Owens, Bethany Morgan Brett, Alison Holmes, Nina Zhu

**Affiliations:** Imperial College London, London, UK; City St George's, University of London, London, UK; Imperial College London, London, UK; Imperial College London, London, UK; My Home Life England, UK; My Home Life England, UK; Imperial College London, London, UK; Imperial College London, London, UK

## Abstract

**Background:**

Rising drug-resistant urinary tract infection (UTI) affects older population disproportionally worldwide, particularly amongst those living in care homes. UTI in care homes were not characterized, and the quality of infection management was not assessed. In this study, we aimed to examine incidence, service and health outcomes of community-acquired UTIs in older adults, comparing those inside and outside care homes.

**Methods:**

We linked and analysed primary, secondary, social care records and pathology data of 2.5 million London population between 2018 and 2023. We matched older adults with UTI in and outside care homes using propensity scores. We performed time-series analysis of UTI incidence rate. We assessed UTI-related outcomes using generalized estimating equations (GEE), adjusted for demographic and other risk factors.

**Results:**

A total of 91 501 UTI cases were analysed; 6648 were from care homes. The incidence rate was 19.1 per 1000 person-months in care homes with an increasing upward trend (increasing by 3.7 per month, *P*=0.014), compared to 5.4 outside while declining (decreasing by 1.3 per month, *P*=0.014). During the COVID-19 pandemic, the UTI incidence increased by further 180.8 episodes per 1000 person- months (*P*=0.004) in care homes, while declining outside by further 25.9 (*P*=0.027). Diagnostic tests, particularly urine culture, were less used in care homes, and the laboratory turnaround was slower. Adherence to diagnostic guidance was lower in care home residents compared to those outside (22.3% versus 45.6%, *P<*0.001). Diagnostic use has stayed persistently low in care home residents even after the resume of face-to-face consultation after the pandemic. UTI in care homes had higher resistance to trimethoprim (46.4% versus 32.0%, *P<*0.001) and nitrofurantoin (7.5% versus 2.4%, P<0.001), and non-susceptibility to ciprofloxacin (29.3% versus 16.4%, *P<*0.001) in *Escherichia coli*, more likely to be polymicrobial, and with significantly worse outcomes within 60 days, measured by the adjusted odd ratio (aOR) of death (1.99, *P<*0.001), complications present to GP (1.38, *P*=0.042) and hospitals (1.15, *P*=0.041), recurrent infection (1.22, *P*=0.001) and antibiotic re-prescription (1.12, *P*=0.010).

**Conclusions:**

Inadequate use of diagnostics and antibiotics led to higher infection burden and worse outcomes in care homes, which was further exacerbated by COVID-19. Targeted infection prevention measures and stewardship interventions are urgently needed to protect the vulnerable population in this setting.

